# Two helices in the third intracellular loop determine anoctamin 1 (TMEM16A) activation by calcium

**DOI:** 10.1007/s00424-014-1603-2

**Published:** 2014-09-19

**Authors:** Jesun Lee, Jooyoung Jung, Min Ho Tak, Jungwon Wee, Byeongjoon Lee, Yongwoo Jang, Hyeyeon Chun, Dong-Jin Yang, Young Duk Yang, Sang Ho Park, Byung Woo Han, Soonsil Hyun, Jaehoon Yu, Hawon Cho, H. Criss Hartzell, Uhtaek Oh

**Affiliations:** 1 Sensory Research Center, Creative Research Initiatives, College of Pharmacy, Seoul National University, Seoul, South Korea; 2 Department of Molecular Medicine and Biopharmaceutical Sciences, Graduate School of Convergence Science and Technology, Seoul National University, Seoul, Republic of Korea; 3 Department of Pharmacy, College of Pharmacy, CHA University, Gyeonggi, South Korea; 4 Department of Pharmacy, College of Pharmacy, Seoul National University, Seoul, Republic of Korea; 5 Department of Chemistry & Education, Seoul National University, Seoul, Republic of Korea; 6 Department of Cell Biology, Emory University School of Medicine, Atlanta, GA USA

**Keywords:** Anoctamin 1, Anoctamin 2, Calcium, Activation, Helix, Structure

## Abstract

Anoctamin 1 (ANO1)/TMEM16A is a Cl^−^ channel activated by intracellular Ca^2+^ mediating numerous physiological functions. However, little is known of the ANO1 activation mechanism by Ca^2+^. Here, we demonstrate that two helices, “reference” and “Ca^2+^ sensor” helices in the third intracellular loop face each other with opposite charges. The two helices interact directly in a Ca^2+^-dependent manner. Positively and negatively charged residues in the two helices are essential for Ca^2+^-dependent activation because neutralization of these charges change the Ca^2+^ sensitivity. We now predict that the Ca^2+^ sensor helix attaches to the reference helix in the resting state, and as intracellular Ca^2+^ rises, Ca^2+^ acts on the sensor helix, which repels it from the reference helix. This Ca^2+^-dependent push-pull conformational change would be a key electromechanical movement for gating the ANO1 channel. Because chemical activation of ANO1 is viewed as an alternative means of rescuing cystic fibrosis, understanding its gating mechanism would be useful in developing novel treatments for cystic fibrosis.

**Electronic supplementary material:**

The online version of this article (doi:10.1007/s00424-014-1603-2) contains supplementary material, which is available to authorized users.

## Introduction

Ca^2+^-activated Cl^−^ channels (CaCCs) mediate trans-epithelial fluid movements and thereby enable secretions in salivary glands, pancreas, intestines, and airways [12, 8, 6]; regulate vascular smooth muscle tone and cardiac myocyte excitability [50, 23, 24]; modulate neuronal cell excitability; and amplify sensory signals in retinal or olfactory sensory neurons [8, 20, 18]. Because of this ability to regulate epithelial secretion, chemical activation of CaCCs is viewed as an alternative means of rescuing disabled fluid movement in the epithelia of cystic fibrosis patients [26, 25, 42].

Anoctamin 1 (ANO1; also known as TMEM16A) is a candidate for Ca^2+^-activated chloride channels and has biophysical and pharmacological properties similar to those of identified CaCCs [47, 3, 31]. As expected for a CaCC candidate gene, ANO1 is expressed in the salivary gland for salivation [47, 30], in the airway epithelium for controlling mucin secretion [13], in the blood vessels for vascular tone [19], and in the pacemaker cells for gastrointestinal smooth muscle contraction [15]. Additionally, ANO1 is known as a heat sensor that mediates thermal pain in sensory neurons [5]. ANO2 is expressed in olfactory sensory neurons, suggesting its role in olfactory signal amplification [2, 35]. ANO2 is also expressed in the hippocampus and controls synaptic excitability [14]. ANO5 is linked to a rare skeletal syndrome, gnathodiaphyseal dysplasia [21, 41, 40]. ANO6 is also known to be a Ca^2+^-activated Cl^−^ channel associated with scramblase activity essential for blood clotting. Its channelopathy is associated with Scott syndrome, a rare disease of hemorrhage [46, 36, 37, 33, 11].

Among the 10 isoforms of anoctamin channel family, ANO1 and ANO2 are activated by physiological concentrations of Ca^2+^ at the resting membrane potential [47, 29]. Despite these multifunctional roles of the anoctamin channel family, the Ca^2+^ activation mechanisms remain elusive. Recently, a putative Ca^2+^-acting site was suggested [48]. Yu and colleagues proposed a revised topology of ANO1 using fluorescent tags at different key positions that differs from the conventional topology of ANO1 [48]. Furthermore, two Glu residues (702-EYME-705) in the third intracellular loop were suggested to be essential for the Ca^2+^ activation. This has been further confirmed by Scudieri and colleagues that a chimera of ANO1 replacing the third intracellular loop with that of ANO2 lowers the Ca^2+^ sensitivity [32]. Although these reports suggest that a region in the third intracellular loop is important for the ANO1 or ANO2 activation by Ca^2+^, molecular insights for the activation is largely unknown. Thus, this study pursues to determine how Ca^2+^ activates ANO1.

## Results

### The third intracellular loop is essential for Ca^2+^-induced ANO1 activation

In order to find potential Ca^2+^-binding sites in the ANO1 sequence, we first measured the Ca^2+^ sensitivities of ANO1 and ANO2. To this end, different concentrations of free Ca^2+^ were applied to the bath of inside-out membrane patches excised from human embryonic kidney (HEK) 293T cells that were transfected with *Ano1* and *Ano2* tagged on the C-terminus with enhanced green fluorescence protein (EGFP). Among splice variants of ANO1, we used an ***a***,***c***-splice variant that has 116 residues in the N-terminus and the 448-EAVK-451 insert [9].

**Fig. 1:**
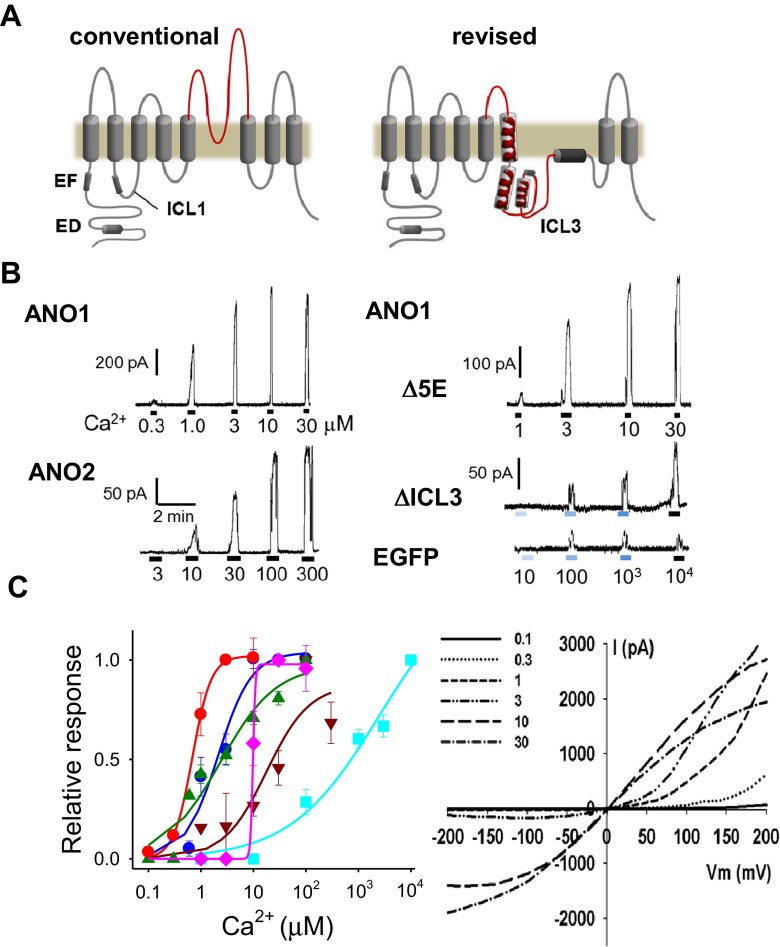
ANO1 activation by Ca^2+^ requires the third intracellular loop (ICL3). **a** Proposed topologies of ANO1, conventional and revised forms. ED, Glu, and Asp-rich region; *EF* EF-hand region; *ICL1* and *ICL3* the first and the third intracellular loops, respectively. **b** Example traces of channel currents of inside-out patches isolated from ANO1, ANO2, ANO1 deletion mutants, and EGFP-transfected HEK 293T cells. *Δ5E* a ANO1 deletion mutant at 444-EEEEE-448, *ΔICL3* a ANO1 deletion mutant of the third intracellular loop region. Holding potential (*E*_h_) = +80 mV. **c** Concentration-response relationships of channel currents of ANO1, ANO2, and ANO1 mutants activated by various concentrations of Ca^2+^. Each current is normalized to the maximal response. *Lines* are fitted to the Hill equation, *I*/*I*max = 1/(1 + [Ca^2+^]/EC_50_)^*n*^. EC_50_s (in μM) for ANO1 (*red circle*), ANO2 (*pink diamond*), and ANO1 mutants were 0.7 (*n* = 11), 9.8 (*n* = 13), 120-H**QNN**KRFRR**QQ**Y**Q**GNLLEAGL**Q**L**Q**ND**Q**DT-148 (ED9 (*green triangle*), 2.4, *n* = 9), Δ5E (*blue circle*, 2.1, *n* = 8), 285-**A**G**A**Y**A**G**A**-291 (*brown inverted triangle*, 17.8, *n* = 6), and ΔICL3 (*light blue square*, 2,509, *n* = 10), respectively. (*Right*) The I/V curves of WT ANO1 at various concentration of Ca^2+^

At +80 mV holding potential (*E*_h_), the application of Ca^2+^ activated ANO1 with a half-maximal concentration (EC_50_) of 0.7 μM (*n* = 11) (Fig. 1b, c). ANO2 was activated by Ca^2+^ with EC_50_ of 9.8 μM (*n* = 13). ANO1 and ANO2 are less sensitive to Ca^2+^ at −80 mV, with respective EC_50_ values of 2.4 (*n* = 10) and 17.9 μM (*n* = 10). Because many mutants of ANO1 and ANO2 failed to respond to Ca^2+^ at −80 mV, thus subsequent experiments were performed with *E*_h_ of +80 mV. In EGFP-transfected cells, small background currents <20 pA were activated by maximal concentration of Ca^2+^ (100 μM ~ 10 mM) (Fig. 1b).

Then, a mutagenesis study was performed to determine a potential Ca^2+^ activation site. ANO1 contains three putative sites that have multiple acidic amino acids. The first site is the Glu- and Asp-rich (E/D rich) region spanning aa121 ~ 147 in the N-terminus. A deletion mutant (aa121 ~ 148) in the E/D rich region was not expressed in the membrane and no currents were observed at a maximal Ca^2+^. However, a mutant having nine Asp or Glu residues replaced with Asn or Gln in the E/D rich region (120-H**QNN**KRFRR**QQ**Y**Q**GNLLEAGL**Q**L**Q**ND**Q**DT-148, ED9) elicited currents with EC_50_ value of 2.4 μM comparable to wild type (Fig. 1c). The second site (285-**D**G**D**Y**E**GDNV**E**FND-297) in the N-terminus shares high homology with the Ca^2+^-binding signature sequence of the EF-hand (**D**x**D**x**D**Gxxxxx**E**) of calmodulin [45, 10]. Replacing all four Asp or Glu residues with Ala shifted the EC_50_ for Ca^2+^ to 17.8 μM (*n* = 6). The third potential Ca^2+^-binding site is located in the first intracellular loop (ICL1) between transmembrane domain 2 and 3 (TM2 and 3). This site has five consecutive Glu residues (444-**EEEEE**AVK**D**HPRA**E**-457), which are loosely aligned with the Ca^2+^-bowl region of large conductance Ca^2+^-activated K^+^ channel [1, 49]. A deletion mutant (444-EEEEE-448) in this region had an EC_50_ of 2.1 μM (*n* = 8) [44] (Fig. 1b, c).

Recently, a revised topology of ANO1 has been proposed in which a loop that was previously thought to be extracellular forms an intracellular loop (Fig. 1a) [48]. A deletion mutant (aa 653–711) of the third intracellular loop (ICL3) in the newly proposed topology was extremely insensitive to Ca^2+^. The dose-response curve was shifted dramatically to the right (EC_50_ = 2.5 mM, *n* = 10) and currents rarely showed a saturation up to 10 mM Ca^2+^ (Fig. 1c). Thus, the ICL3 region appears important for activation of ANO1 by Ca^2+^.

### Structural prediction of the ICL3 region

**Fig. 2:**
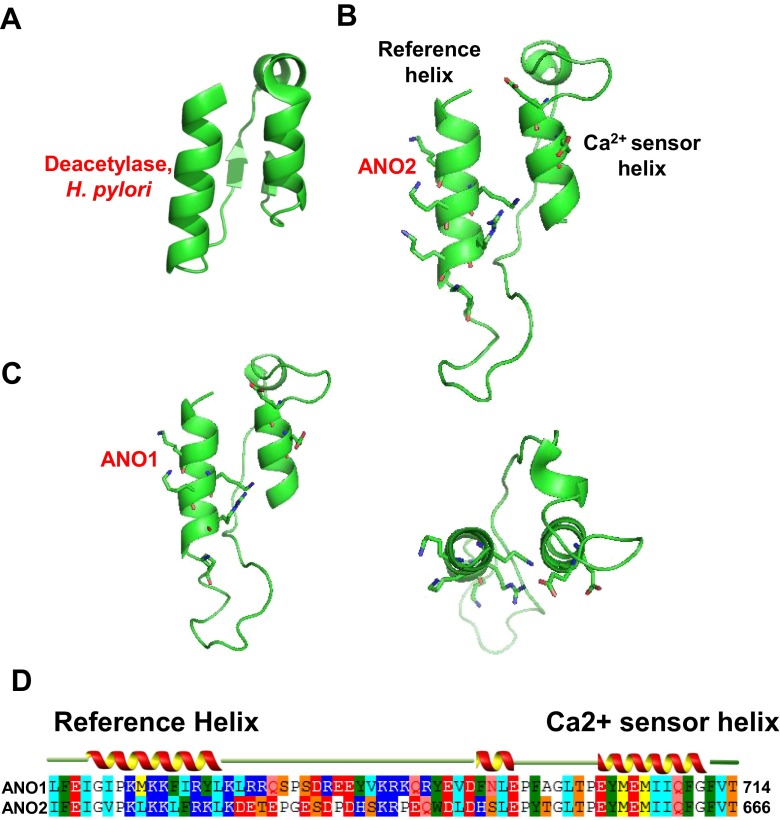
Homology models of the ICL3 regions of ANO1 and ANO2. **a** A crystal structure of a part of a peptidoglycan deacetylase of *Helicobacter pylori* that shares structural homology with the ICL3 region of ANO2. **b** A homology model of the ICL3 of ANO2. Structure of the ICL3 region of ANO2 was modeled using the backbone coordinates of a deacetylase of *Helicobacter pylori* as a template. Side chains of Arg and Lys are shown in *stick. Blue* and *red atoms* in the side chains represent nitrogen and oxygen atoms, respectively. **c** A homology model of the ICL3 of ANO1. Structure of the ICL3 region of ANO1 was modeled using the backbone coordinates of the ICL3 region of ANO2 as a template, (*right*) top view. (**d**) Sequence alignment of the ICL3 regions of ANO1 and ANO2

In an attempt to glean insights into the structure of this region, we searched for crystal structures homologous to the ICL3 segment from the protein MODWEB server, an automated homology-modeling program (https://modbase.compbio.ucsf.edu/modweb/) that uses MODPIPE, an automated program for protein structural modeling that selects homologous structures from a large number of identified crystal structures of proteins [7, 28]. This search revealed that a partial segment (residues 606 ~ 663) of the ICL3 of ANO2 is homologous to a part of the crystal structure (PDB ID: 3QBU) of a peptidoglycan deacetylase of *Helicobacter pylori*, with 31.6 % sequence identity (Fig. 2a, b and Supplementary Fig. 1). The model was considered reliable because the model satisfied GA341 (0.78, the score based on statistical potentials higher than the pre-specified cutoff; 0.7) and z-DOPE (−0.6, an atomic distance-dependent statistical potential from a sample of native structures, reliable if z-DOPE <0) [7]. Structure-homology modeling (PyMOL) of the ICL3 segment of ANO2 showed two parallel α-helices in close proximity (Fig. 2b), one of which is the Lys- and Arg-rich helix. Interestingly, the other helix contains the two Glu residues that had been considered important for Ca^2+^ sensitivity in ANO1 [48] (Fig. 2b). Because these two Glu residues appear critical for Ca^2+^-induced activation, we called this helix the “Ca^2+^ sensor helix.” The counterpart helix that appears important for interacting with the Ca^2+^ sensor helix was called the “reference helix.” The two helices were linked by 23 amino acids (Fig. 2d). We also modeled an ANO1 structure (amino acids 653–711) using the backbone of the ANO2 ICL3 region as a template. Overall, the structure of ANO1 resembled that of ANO2 (Fig. 2c). When the reference helix regions were aligned, ANO1 and ANO2 had four and five basic residues, respectively.

### Gating mechanism by Ca^2+^

Because the two parallel helices in the ICL3 region of ANO1 have amino acids with opposite charges, these opposing charges may engage in the activation of ANO1 by Ca^2+^. We predicted that, in the closed state, the two helices hold together primarily due to the ionic interactions between positive and negative charges in the two helices (Fig. 7f). However, as intracellular Ca^2+^ increases, Ca^2+^ binds and covers the Ca^2+^ sensor helix around the two Glu residues. These positive charges from the Ca^2+^ sensor helix may break the ionic interaction between the two helices and repel the reference helix away from the Ca^2+^ sensor helix (Fig. 7f). When the Ca^2+^ ions are removed, the Ca^2+^ sensor helix again moves closer to the reference helix. This conformational change of the two helices would lead to a conformational change in the channel gate.

### Ca^2+^-dependent interaction of the reference and Ca^2+^-sensor helices

**Fig. 3:**
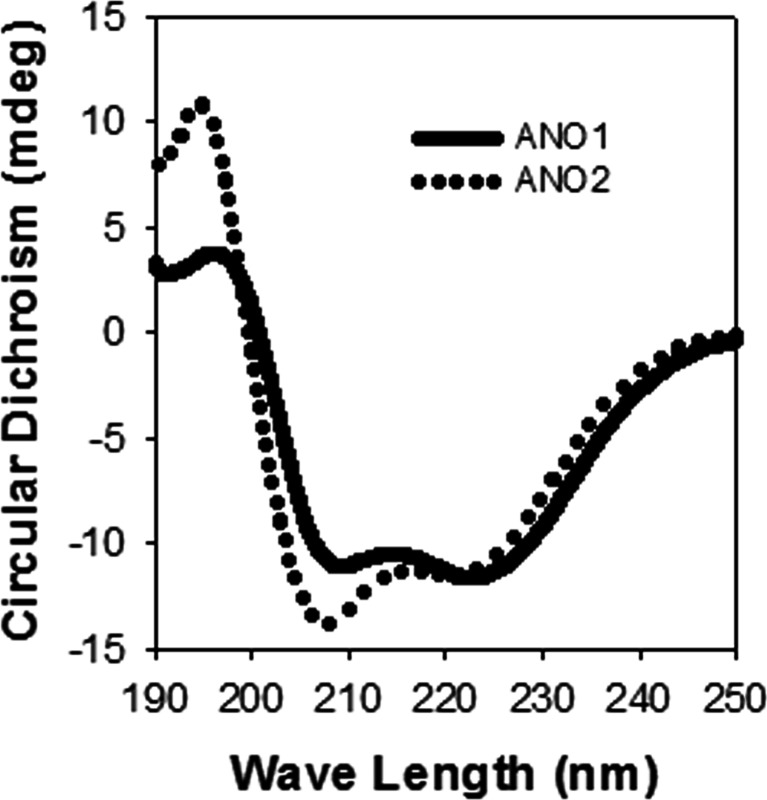
Circular dichroism spectra of two reference helix peptides of ANO1 and ANO2. The two peptides that span the reference helices in the ICL3 regions of ANO1 and ANO2 were 652-LFEIGIPKMKKFIRYLKL-669 and 604-IFEIGVPKLKKLFRKLKD-621, respectively. Note a typical α-helical character with a positive band around 192 nm and negative bands around 208 and 222 nm

To confirm whether the reference helix of ANO1 forms an α-helix, circular dichroism spectroscopy was performed with synthetic peptides spanning the reference helix regions of ANO1 (652-LFEIGIPKMKKFIRYLKL-669) and ANO2 (604-IFEIGVPKLKKLFRKLKD-621). The two peptides of ANO1 and ANO2 had a typical circular dichroism spectrum of an α-helix with a positive band around 192 nm and negative bands around 208 and 222 nm (Fig. 3) [34].

**Fig. 4:**
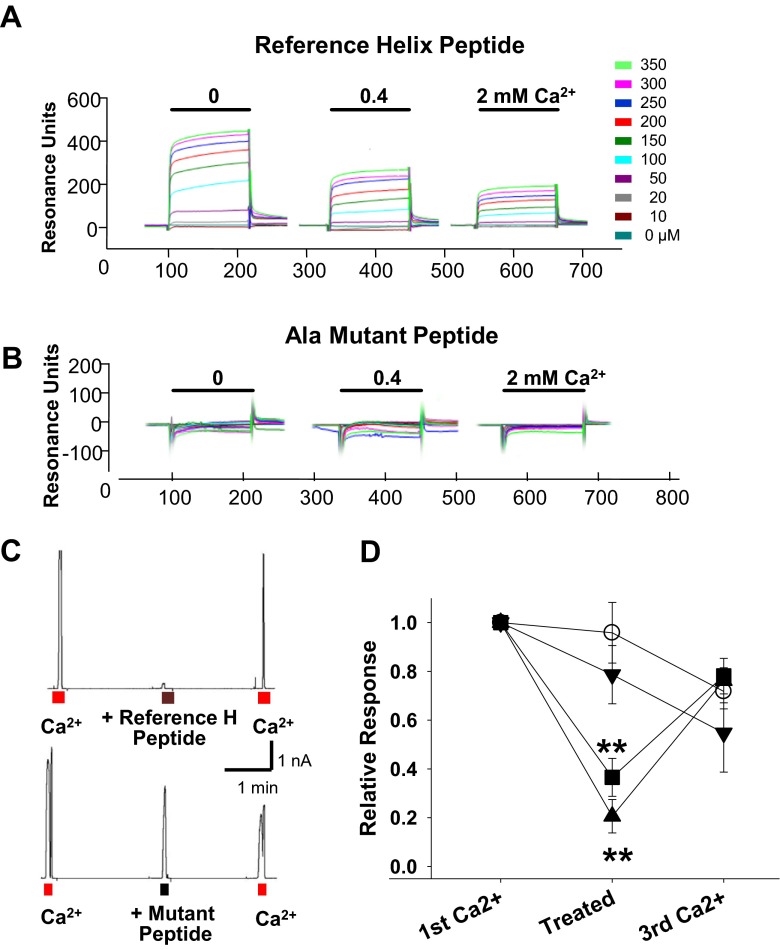
The reference helix of the ICL3 region interacts with the Ca^2+^ sensor helix in a Ca^2+^-dependent way. **a**, **b** Physical interaction between the reference and Ca^2+^ sensor helices was assessed with a surface plasmon resonance assay. Biotinylated Ca^2+^ sensor helix peptide (692-NLEPFAGLTPEYMEM-706) was immobilized on a streptavidin-coated gold sensor chip. Resonance intensities were measured after solutions containing 0, 0.4, and 2.0 mM Ca^2+^ and various concentrations of reference helix peptide (651-NLFEIGIP**K**M**KK**FI**R**YL**K**L**RR**-671) (**a**) or its Ala-substituted mutants (651-NLFEIGIP**A**M**AA**FI**A**YL**A**L**AA**-671) (**b**) were flowed through the gold chip. Note that the wild-type reference helix peptide shows a binding with the Ca^2+^ sensor helix in a Ca^2+^-dependent manner (**a**), which is not observed for the Ala-substituted mutant peptide (**b**). **c** Ca^2+^-induced ANO1 currents were blocked by bath application of the reference helix peptide (651-NLFEIGIP**K**M**KK**FI**R**YL**K**L**RR**-671, 4 μM) to inside-out patches but not by the Ala-mutant peptide (651-NLFEIGIP**A**M**AA**FI**A**YL**A**L**AA**-671, 4 μM) (*lower panel*). Ca^2+^ (3 μM) was applied three times to inside-out patches of ANO1-transfected HEK cells. At the second Ca^2+^ challenge, the peptides were also given. *E*_h_ = +80 mV. **d** A summary plot of the effects of applications of reference helix and Ca^2+^ sensor helix peptides on Ca^2+^-activated ANO1 currents. Current amplitudes were normalized to the current amplitude obtained after of the first Ca^2+^ challenge. At the second Ca^2+^ challenge, reference helix peptide (*black circle*, **p < 0.01 compared to the relative response of vehicle application, one-way ANOVA, Newman-Keuls post hoc test, *n* = 13), Ala-replaced reference helix peptide (*black inverted triangle*, *n* = 8), the Ca^2+^ sensor helix peptide (700-TPEYMEMIIQFGF-712) (*black square*, **p < 0.01 compared to the relative response of vehicle application, one-way ANOVA, Newman-Keuls post hoc test, *n* = 7), and vehicle (*white circle*, *n* = 8) were also applied

According to the gating hypothesis, physical binding of the two helices and the interference of Ca^2+^ in this interaction are key determinants for gating ANO1. To investigate this relationship, physical interaction between the two helices was measured using surface plasmon resonance. A peptide segment (692-NLEPFAGLTPEYMEM-706) spanning the Ca^2+^ sensor helix of ANO1 was synthesized, biotinylated at the N-terminus, and immobilized on a streptavidin-coated gold sensor chip. Subsequently, a synthetic peptide spanning the reference helix (651-NLFEIGIP**K**M**KK**FI**R**YL**K**L**RR**-671) was passed over the immobilized Ca^2+^ sensor helix. In the absence of Ca^2+^, the reference helix peptide interacted strongly with the Ca^2+^ sensor helix (Fig. 4a). Ca^2+^ (0.4–2 mM) produced a dose-dependent reduction in the interaction between the two synthetic helix peptides. In contrast, a mutant of the reference helix peptide (651-NLFEIGIP**A**M**AA**FI**A**YL**A**L**AA**-671) in which all Lys and Arg residues were replaced with Ala failed to show any interaction with the Ca^2+^ sensor helix peptide regardless of Ca^2+^ concentration (Fig. 4b). These results clearly indicate that the two helices interact directly with each other in a Ca^2+^-dependent manner.

We then applied the reference helix and Ca^2+^ sensor helix peptides to isolated inside-out patches of HEK 293T cells transfected with ANO1, to see if the peptides antagonized Ca^2+^ in activating ANO1. Indeed, application of 4-μM reference helix peptide or 10-μM Ca^2+^ sensor helix peptide (700-TPEYMEMIIQFGF-712) markedly blocked Ca^2+^-induced ANO1 currents (Fig. 4c, d). In contrast, the Ala-mutant reference helix peptide failed to block the Ca^2+^-induced ANO1 currents.

### Mutations in the two helices of ICL3 alter the Ca^2+^ sensitivity

**Fig. 5:**
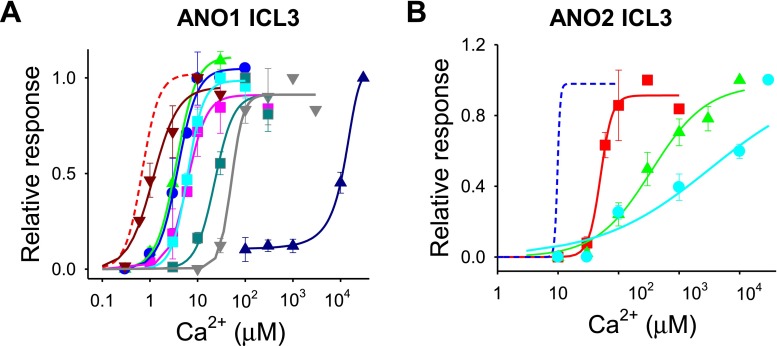
Mutations in the ICL3 regions of ANO1 and ANO2 shift the sensitivity of Ca^2+^-dependent activation. **a** Replacement of Arg, Lys, or Glu residue with Ala, Gly, or Gln residue in the reference and Ca^2+^ sensor helices caused rightward shifts in the dose-response relationship curve of ANO1. EC_50_s (in μM) for ANO1 mutants were 659-**Q**M**KK**-662 (*pink square*, 6.1, *n* = 4), **K**M**QK** (*blue circle*, 4.7, *n* = 5), **K**M**KQ** (*green triangle*, 3.6, *n* = 6), **G**M**GG** (*dark green square*, 22.8, *n* = 6), 665-**G**YL**G**-668 (*light blue square*, 6.2, *n* = 6), deletion of 659-**K**M**KK**FI**R**YL**K**-668 (*gray inverted triangle*, 51.0, *n* = 9), 670-**GG**Q-672 (*brown inverted triangle*, 1.2, *n* = 7), and 702-**Q**YM**Q**-705 (*blue square* 11260, *n* = 7). The dose-response curve of wild-type (WT) ANO1 is the same as shown in Fig. 1c (*red line*). **b** Mutations in the ICL3 region of ANO2 also shifted dose-response curves rightward. EC_50_s were 611-**Q**L**QQ**-614 (*red square*, 50.2 μM, *n* = 7), 611-**G**L**GG**LF**GG**L**G**-620 (*green triangle*, 348.9 μM, *n* = 8), and 654-**A**YM**A**-657 (*light blue circle*, 3.3 mM, *n* = 6). The dose-response curve of WT ANO2 (*blue line*) is same as shown in Fig. 1c

To determine whether charged residues in the two helices are essential for the ANO1 activation by Ca^2+^, we constructed ANO1 and ANO2 mutants whose Arg or Lys residues in the reference helix were replaced with non-charged residues (Ala, Gln, or Gly). Replacement of single Lys residues to Gln in the upper part of the reference helix such as 659-**K**M**KK**-662 mutant caused a rightward shift of EC_50_ values, ranging from 3.6 to 6.1 μM (Fig. 5a). When all three Lys residues in the region were replaced with Gly (659-**G**M**GG**-662), the EC_50_ increased to 22.8 μM (*n* = 6). Replacement of Arg or Lys residues with Gly in the lower part of the reference helix such as 665-**G**YL**G**-668 mutant shifted the EC_50_ value to 6.2 μM (*n* = 6). Furthermore, deletion (ΔKMKKFIRYLK) of the reference helix changed the EC_50_ values to 51.0 μM (*n* = 9). A mutation of the two Glu residues in the Ca^2+^ sensor helix (702-**E**YM**E/Q**YM**Q**-705) elicited a four-order magnitude of rightward shift in EC_50_ (11.3 mM, *n* = 7) (Fig. 5a). Likewise, mutations in the reference helix of ANO2 also reduced the potency of Ca^2+^ in opening ANO2. Replacing all three Lys residues to Gln in the upper part of the reference helix increased the EC_50_ from 9.8 to 50.2 μM. In addition, replacement to Gly of all charged residues in the reference helix (611-**G**L**GG**LF**GG**L**G**-620) caused a dramatic rightward shift of EC_50_ (349 μM, *n* = 8) (Fig. 5b). Mutation of the two Glu residues in the Ca^2+^ sensor helix (654-**E**YM**E/A**YM**A**-657) elicited a three-order magnitude of rightward shift in EC_50_ (3.3 mM, *n* = 6) (Fig. 5b).

### E_act_ acts on the reference helix peptide

**Fig. 6:**
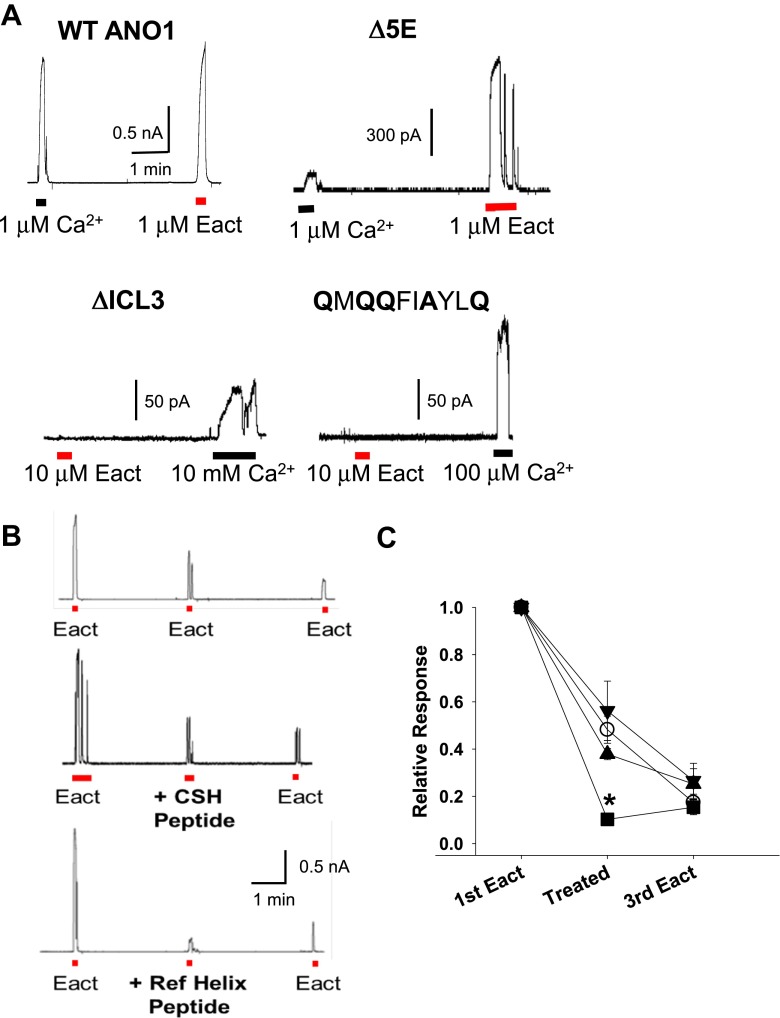
E_act_, a synthetic agonist of ANO1 acts on the ICL3 region. **a** Example traces depicting activation by E_act_ of WT ANO1 (*upper left*), 444-EEEEE-448 (Δ5E, *upper right*), ΔICL3 (*lower left*), and 659-**Q**M**QQ**FI**A**YL**Q**-668 mutant (*lower right*). **b** Example traces of E_act_-induced ANO1 currents, which were blocked by bath application of the reference helix peptide (Ref helix peptide, 651-NLFEIGIPKMKKFIRYLKLRR-671, 4 μM, *lower panel*) but not by the Ca^2+^ sensor helix peptide (CSH peptide, 700-TPEYMEMIIQFGF-712, 10 μM, *middle panel*). To test the effects of peptide, 1 μM E_act_ was applied three times. At the second E_act_ challenge, each peptide was also applied. **c** A summary of the effects of applications of helix peptides on E_act_-induced ANO1 currents. Current amplitudes were normalized to the current amplitude obtained after of the first E_act_ challenge. Reference helix peptide (*black square*, *n* = 5), its Ala-mutant peptide (*black inverted triangle*, *n* = 5), Ca^2+^ sensor helix peptide (*black triangle*, *n* = 5), or vehicle (*white circle*, *n* = 14) was applied at the second E_act_ challenge. *p < 0.05 compared to the relative response of vehicle application, one-way ANOVA followed by Newman-Keuls post hoc test

E_act_, a synthetic agonist of ANO1, was synthesized for the purpose of treating cystic fibrosis in an attempt to bypass dysfunctioning CFTR channel [22]. Even though E_act_ was known to activate ANO1, the activation mechanism is not known. We therefore hypothesized that E_act_ also acts on the ICL3 region. Application of 1 μM E_act_ to the bath of inside-out patch activated ANO1 (Fig. 6a). In addition, maximal E_act_ (10 μM) activated the Δ5E ANO1 mutant that deleted the Ca^2+^ bowl-like region in ICL1 (Fig. 6a). E_act_ also activated 285-**A**G**A**Y**A**G**A**-291 mutant that replaced acidic amino acids in the EF-hand-like region in the N-terminus (data not shown). However, when applied to ΔICL3, the deletion mutant of ANO1 in the ICL3 region, E_act_ failed to activate, whereas 10 mM Ca^2+^ evoked a small current (Fig. 6a). In addition, E_act_ failed to activate both the **Q**YM**Q** (data not shown) and 659-**Q**M**QQ**FIAYL**Q**-668 mutants that neutralized charges in the Ca^2+^ sensor helix and reference helix, respectively (Fig. 6a). These results suggest that E_act_ acts on the ICL3 region.

We then applied the two helix peptides to see if these peptides compete with E_act_ in activating ANO1. When 10 μM Ca^2+^-sensor helix peptide was applied together with 1 μM E_act_ to inside-out patches from ANO1-transfected cells, the peptide failed to inhibit E_act_-activated currents (Fig. 6b, c). In contrast, when 4-μM reference helix peptide was applied together with E_act_, the peptide significantly inhibited the E_act_-activated ANO1 currents (*p* < 0.05, one-way ANOVA, Newman-Keuls post hoc test, *n* = 5 ~ 14). However, the Ala-substituted reference helix peptide failed to inhibit the E_act_-activated ANO1 currents (Fig. 6c). These results clearly suggest that E_act_ acts on the reference helix in the ICL3 region.

### The two helices in the ICL3 region are dispensable for voltage- and heat-induced ANO1 activation

**Fig. 7:**
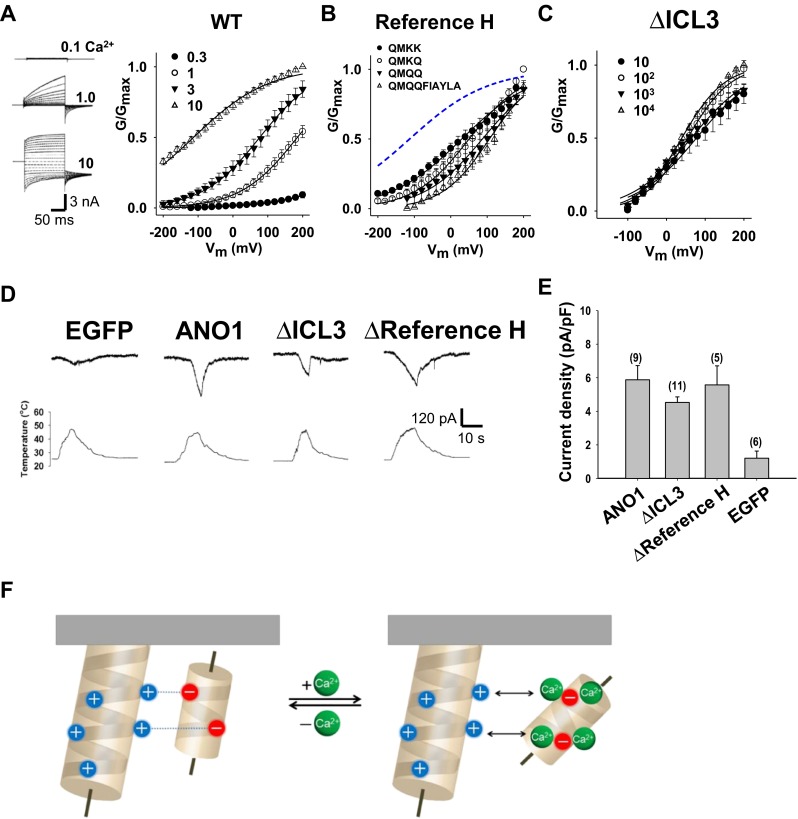
The two helices in the ICL3 region are dispensable for voltage- and heat-induced ANO1 activation. **a** Current responses of WT ANO1 to voltage pulses (−200 to +200 mV in 20-mV increment) at 0.1, 1.0, and 10 μM Ca^2+^ (*left*). A G–V curve of WT ANO1 at 0.3, 1.0, 3.0, and 10 μM Ca^2+^ (*right*). G/G_max_s at different [Ca^2+^]_*i*_ were plotted against membrane potential (*V*_m_). **b**, **c** G–V curves of various mutants in the reference helix at 10 μM [Ca^2+^]_*i*_ (**b**) and an ICL3-deleted mutant (residues 653–711) of ANO1 at 10 μM ~ 10 mM [Ca^2+^]_*i*_ (**c**). The G–V curve of WT ANO1 was the same as shown in Fig. 7a (*black line*). **d** Traces of heat-induced whole-cell currents of EGFP-, WT-, ΔICL3-, and Δreference helix mutant (Δ659-668)-transfected HEK 293T cells. *E*_h_ = −60 mV. **e** A summary of heat-induced currents of ANO1 and its mutants. **f** A schematic diagram depicting a molecular mechanism underlying Ca^2+^-dependent activation of ANO1 at the ICL3 region

We reasoned that the positively charged residues in the reference helix may play a role in mediating voltage-dependent activation. Progressive replacement of Lys residues in the upper part of the reference helix shifted the half-maximal activation voltages (V_1/2_) in G–V curves, an indication of voltage-independent activation, from −112 to +106 mV without changing the slopes (*z* = 0.24 ~ 0.39) of the G–V curve (Fig. 7b). When the ICL3 region of ANO1 (residues 653 ~ 711) was deleted, the V_1/2_s of the G–V curves did not vary (+39.5 ~ +77.3 mV) between 10 μM and 10 mM [Ca^2+^]_i_ (Fig. 7c), which contrasts to a 294-mV change in V_1/2_ of the wild-type ANO1 (Fig. 7a). In addition, the slopes of G–V curves did not vary (*z* = 0.32 ~ 0.46) upon deletion; the ICL3 region is not a site for voltage-induced activation.

Another activation stimulus for ANO1 is heat. Bath temperature greater than 44 °C is sufficient to open the ANO1 channel [5]. Because the activation by Ca^2+^ and heat are synergistic, we tested whether the heat-induced ANO1 activation is also mediated by the ICL3 region. To eliminate activation by Ca^2+^, we removed Ca^2+^ from the pipette solution (0 Ca^2+^ with 5 mM ethylene glycol tetraacetic acid (EGTA)) in whole-cell recording. Surprisingly, a mutant (ΔICL3) which hardly responded to sub-millimolar Ca^2+^ concentration elicited currents when the temperature of bath solution was raised over 44 °C (Fig. 7d, e). Thus, these results suggest that the activation mechanism of ANO1 by heat is different from that by Ca^2+^.

## Discussion

With homology modeling, we predict that the ICL3 region in the revised topology of ANO1 has a unique structure composed of two parallel helices that interact with each other in a Ca^2+^-dependent manner and cause rightward shifts in the Ca^2+^ sensitivity upon mutation. Furthermore, E_act_, a synthetic agonist of ANO1, also acts on the ICL3 region. However, the two helices apparently do not control voltage-induced activation because mutagenesis did not change the sensitivity to voltage. Furthermore, the ICL3 region does not account for heat-induced activation. Although this study is based on homology modeling, present experimental data including surface plasmon resonance (SPR) assay, circular dichroism analysis, as well as mutational study along with electrophysiological experiments support the structure model.

According to the model of ICL3 of ANO1, several structural elements appear important for the activation by Ca^2+^. The two helices are oppositely charged. The role of positive charges in the reference helix appears to hold the Ca^2+^ sensor helix in the resting state. We imagine that the reference helix pushes the Ca^2+^-sensor helix away when Ca^2+^ ions attach to it (Fig. 7f). Thus, removal of these positive charges in the reference helix decreases the direct binding and sensitivity to Ca^2+^ concentrations. However, because the removal of positive charges in the reference helix showed two-order differences in Ca^2+^ sensitivity that is smaller than the four-order change with the mutant in the Ca^2+^ sensor helix (Fig. 5a), factors other than the ionic interaction may also contribute to the movement of two helices by Ca^2+^. In line with this, positively charged amine residues of Lys and Arg in the reference helix do not align along one side facing the Ca^2+^ sensor helix. Instead, these positive charges distribute in many directions in a circular fashion (Fig. 3). In addition, only ANO1 and ANO2 are sensitive to physiological range of Ca^2+^ at the resting membrane potential and have the helix-loop-helix structure in the ICL3 region. It is uncertain whether other ANO family members have this structural element. It is conceivable that these other ANO channels may have different mechanisms for channel gating if they are channels. However, as ANO6 has been suggested as a scramblase [46, 36], other ANO family members may function other than channels.

Many studies tried to define the Ca^2+^ activation site in ANO1. Initially, the Ca^2+^ bowl-like region in the ICL1 was studied for the Ca^2+^ sensitivity [44]. However, the deletion of the region failed to show a shift in the Ca^2+^ sensitivity. The lack of change in the Ca^2+^ sensitivity of mutants in this region of ANO1 and ANO2 was also observed by others [4]. Furthermore, Tien and colleagues observed that an “***a***” variant of ANO1 lacking the “EAVK” residues retains the EC_50_ of Ca^2+^ less than 1 μM, comparable to that of the “***a***,***c***” variant [38]. Similarly, we also failed to see a discernible change in EC_50_ after the deletion of 444-EEEEE-448 (Fig. 1b, c). In contrast, Xiao and Cui reported that the deletion of 444-EEEEEAVK-451 shifted ~40-fold increase in EC_50_ [43]. Yu and colleagues found the acidic residues, E702 and E705, in the ICL3 region of the revised topology that were critical for the Ca^2+^ sensitivity of ANO1 [48]. Scudieri and colleagues also confirmed that this region is sensitive to the Ca^2+^-dependent activation [32]. The present study also confirmed that this region is important for the ANO1 activation by Ca^2+^. Recently, Tien and colleagues also suggest that the ICL3 region is an important site for the Ca^2+^ sensitivity [38]. They extended mutation to E734 (equivalent to E730 in “***a***” variant) and D738 (equivalent to D734 in “***a***” variant) residues beyond the E702 and E705 residues and found that the E734 and D738 are involved in the Ca^2+^-dependent gating, because mutations in these residues made a large shift in EC_50_. Thus, Tien and colleagues proposed that the Ca^2+^ caging with the four Glu and Asp residues is essential for the ANO1 activation. It is not clear how Ca^2+^ binding to this site gates ANO1. It is conceivable that these additional Glu734 and Asp738 residues may affect the interaction between Ca^2+^ and the reference Ca^2+^ sensor helices, because the two Glu and Asp residues are close to the Ca^2+^ sensor helix. However, to identify the interactions between the two helices and the E734 and D738 residues requires further study.

In the present study, high Ca^2+^ evoked currents in ΔICL3 mutant-expressing HEK cells. This remnant current is probably caused by endogenous ANO isoforms present in native HEK cells. HEK cells are known to have ANO6 and 8 [27]. Thus, the current activated by high Ca^2+^ in ΔICL3 mutant-expressing cells would be currents of endogenous ANO6 or other isoforms in HEK cells. Another possibility is that there are multiple sites other than the ICL3 region in ANO1 that controls the Ca^2+^-dependent activation. So, the deletion of the ICL3 region may not remove all the currents activated by Ca^2+^.

In summary, this study demonstrates the structural basis of channel gating by Ca^2+^ and voltage in ANO1. Although electrophysiological, protein chemistry, and structure modeling have predicted that opposite charges between the two helices in the ICL3 play critical roles in channel gating, the precise mechanism underlying the activation requires crystal structure analysis. Because ANO1 and other family members mediate numerous physiologic functions, elucidation of the activation mechanism by Ca^2+^ is essential for a detailed understanding of ANO-related pathophysiology. ANO1 agonists are now considered to be useful in the management of cystic fibrosis. Understanding structural and molecular mechanisms for ANO1 activation is useful in designing anti-cystic fibrosis drugs.

## Materials and methods

### Mutagenesis and gene expression

All mutants were generated from the wild-type construct, mouse ANO1 (pEGFP-N1-mANO1). Amino acid substitution or deletion mutants were prepared using a site-directed mutagenesis kit (Muta-direct, iNtRON Biotech) or by using the overlap PCR method. Mutations in all mutants were confirmed by sequencing whole nucleotide sequences. HEK 293T cells were transfected with 1 μg of pEGFP-N1-mANO1 or pEGFP-N1-mutants, 0.05 μg pEGFP-N1, and FuGENE (Roche Diagnostics, Penzberg, Germany) in 35-mm dishes. Transfected cells were incubated in DMEM supplemented with 10 % fetal bovine serum (GIBCO) and penicillin-streptomycin at 37 °C in a 5 % CO_2_ incubator. Cells were used 1 or 2 days after transfection.

### Electrophysiology

Borosilicate glass pipettes (World Precision Instruments, Sarasota, FL) with tip resistances of ~2 Mohms were used to form gigaseals on HEK cells. Inside-out membrane patches were excised by pulling the pipette away from the cell. Currents were recorded with a patch-clamp amplifier (Axopatch 200B, Molecular Probes) with a 1-KHz filter. Data from the amplifier were digitized with Digidata 1440A (Molecular Probes) and stored on a computer.

The control pipette solution contained 140 mM N-methyl d-glucamine, 2 mM MgCl_2_, and 10 mM HEPES adjusted to pH 7.2 with HCl. For Ca^2+^-free solution, 10 mM ethylene glycol tetraacetic acid (EGTA) was added to the control solution. The bath solution contained (in mM) 140 N-methyl d-glucamine, 2 MgCl_2_, 10 HEPES, 10 chelator (EGTA, N(2-hydroxyethyl) ethylenediamine triacetic acid (HEDTA), or nitrilotriacetic acid (NTA)), and a calculated amount of CaCl_2_ (adjusted with HCl to pH 7.2). EGTA was used for 0.1 ~ 1.0 μM free Ca^2+^, whereas HEDTA and NTA were used for 3.0 ~ 30 and 100 ~ 1,000 μM free Ca^2+^, respectively [39]. No chelator was added to solutions with free Ca^2+^ greater than 1 mM. To calculate free Ca^2+^ in pipette solutions, the WEBMAXC program was used (http://www.stanford.edu/~cpatton/webmaxc/webmaxcS.htm). Free Ca^2+^ less than 30 μM in each solution was monitored with Fura-2 fluorometry [17].

### Surface plasmon resonance measurement

A Biacore 3000 was used for kinetic surface plasmon resonance measurement as previously described [16]. Briefly, a biotinylated peptide (692-NLEPFAGLTPEYMEM-706) was immobilized on a SA chip or custom streptavidin-coated CM5 sensor chip (GE Healthcare), with one flow cell with biotin alone as a reference cell. For the binding assay, HEPES-buffered saline containing 0.005 % of Tween 20 was used as a running buffer. Various concentrations of the reference helix peptide (aa 651–671) were injected for 120 s and dissociated for 240 s at a flow rate of 20 μL/min. Binding curves were analyzed using BIAevaluation 3.1.

### Structure homology modeling

The amino acid residues of third intracellular loop (residues 603–663) in ANO2 were selected as the target sequence for homology modeling. Automated homology modeling was implemented by MODWEB (http://salilab.org/modweb), a web server for automated comparative protein structure modeling.

## Electronic supplementary material

Below is the link to the electronic supplementary material.

Supplementary Fig. 1 Crystal structure of a peptidoglycan deacetylase of Helicobacter pylori. The ICL3 region of ANO2 (green) is overlaid (PPTX 170 kb)
