# Supplementary figures and images for: Two helices in the third intracellular loop determine anoctamin 1 (TMEM16A) activation by calcium
Source: Pflugers Arch. 2014 Sep 19;467(8):1677–87. doi: 10.1007/s00424-014-1603-2 (PMC4502317; doi:10.1007/s00424-014-1603-2)

## Slide 1
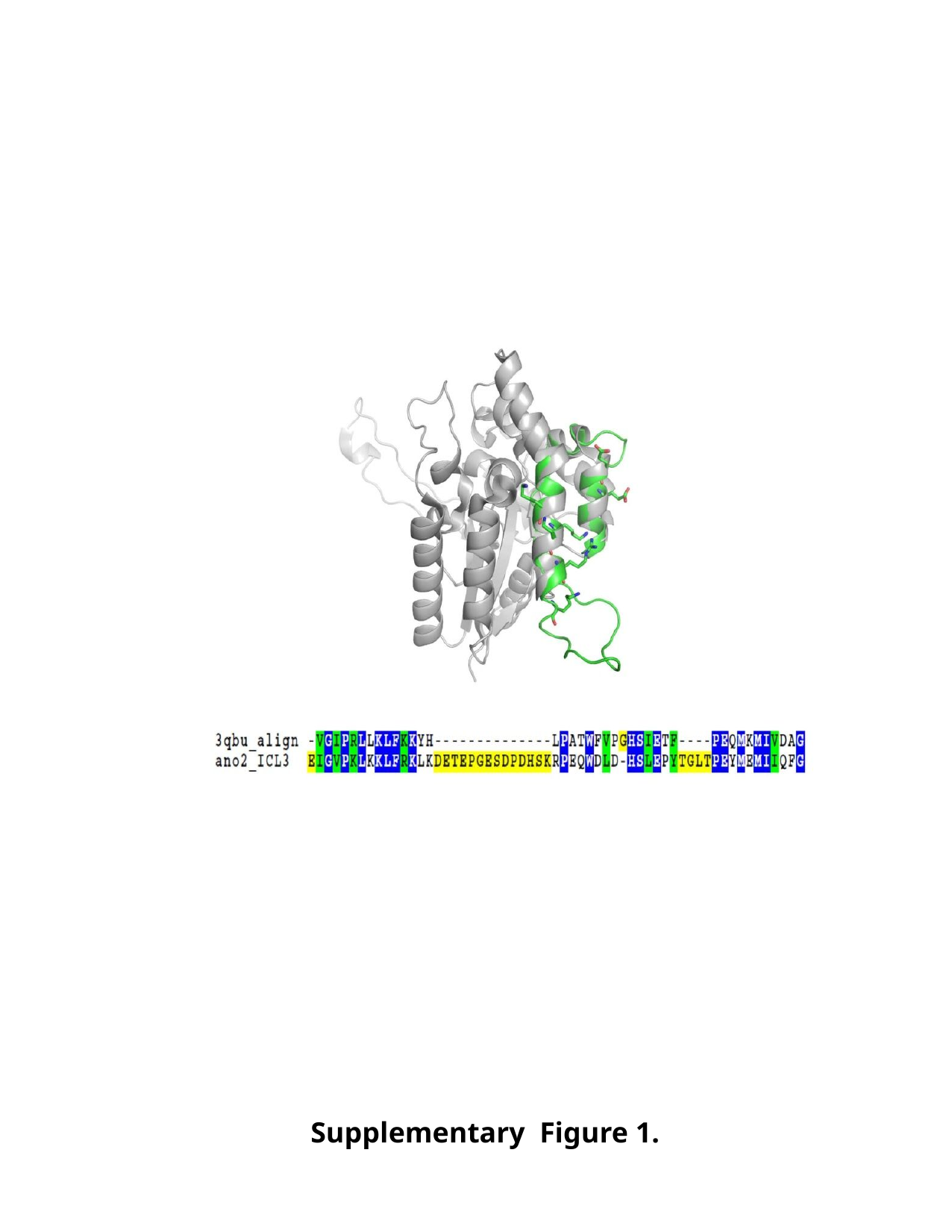

Supplementary Figure 1.

Supplement: Supplementary file 1 — Crystal structure of a peptidoglycan deacetylase of Helicobacter pylori. The ICL3 region of ANO2 (green) is overlaid (PPTX 170 kb) [file 424_2014_1603_MOESM1_ESM.pptx]
